# Connaissances et perceptions des personnes vivant avec le VIH sur les dispositifs d'aide à l'observance dans les centres de traitement de la région du Plateau-Central du Burkina Faso, novembre 2024

**DOI:** 10.48327/mtsi.v5i2.2025.643

**Published:** 2025-05-12

**Authors:** Wedminère Noélie ZOUNGRANA-YAMEOGO, Dominique Hélène Laurel YABRE, Fidèle BAKIONO, Toussaint COMPAORE, Arielle Rita BELEM, David KANGOYE, Christian Philippe YONLI, Ouo Mireille COULIBALY, Koiné Maxime DRABO

**Affiliations:** 1 Département de santé publique, Centre hospitalier universitaire de Tengandogo, Ouagadougou, Burkina Faso; 2 Institut de formation de la recherche interdisciplinaire en science de la santé et de l’éducation; 3 Secrétariat permanent du Conseil national de lutte contre le Sida, Ouagadougou, Burkina Faso; 4 Direction régionale de la région du Plateau-Central, Burkina Faso; 5 Service de maladies infectieuses, Centre hospitalier universitaire de Tengandogo, Ouagadougou, Burkina Faso; 6 Institut national de santé publique, BP 10278 OUAGA Ouagadougou, Burkina Faso; 7 Institut de recherche en sciences de la santé, Ouagadougou, Burkina Faso

**Keywords:** VIH, PvVIH, Enquête CAP, Dispositifs antirétroviraux, Observance, Burkina Faso, Afrique subsaharienne, HIV, PLHIV, KAP survey, Antiretroviral drugs, Adherence, Burkina Faso, Sub-Saharan Africa

## Abstract

**Introduction:**

Au Burkina Faso, depuis l'introduction de la thérapie antirétrovirale contre le virus de l'immunodéficience humaine (VIH), plusieurs dispositifs d'aide à l'observance au traitement ont été mis en place pour améliorer les résultats du traitement et prévenir la résistance. L'objectif de notre étude était d'évaluer les connaissances et les perceptions des personnes vivant avec le VIH (PvVIH) sur ces dispositifs d'aide à l'observance thérapeutique existant dans la région du Plateau-Central.

**Méthodes:**

Nous avons mené une étude descriptive dans la région du Plateau-Central, l'une des 13 régions du Burkina Faso. Les PvVIH ont été sélectionnées au fur et à mesure qu'elles venaient à leur visite de suivi. Les informations ont été collectées par entretien à partir d'un questionnaire standardisé. L'observance a été calculée sur la base des déclarations des personnes. Les personnes qui avaient pris totalement leurs médicaments le mois précédent l'enquête étaient considérées comme observantes. Les variables quantitatives ont été calculées en utilisant la moyenne et les variables qualitatives la proportion.

**Résultats:**

Au total, 347 PvVIH ont été prises en compte dans l’étude. Parmi elles, 69 % étaient des femmes, l'âge moyen était de 45,6 ± 12,2 ans. La durée moyenne de suivi du traitement était de 8,6 ± 5 ans. La proportion des personnes observantes au traitement était de 80 % IC95 % [75-84]. Presque toutes les PvVIH (99,7 %) connaissaient l'existence des dispositifs d'aide à l'observance. Les principaux dispositifs connus étaient l'approvisionnement semestriel en médicaments antirétroviraux (RAVI6M) (71 %), les groupes de discussion (69,9 %), l'entretien avec la personne (69,9 %) et le *counseling* (64,2 %). Le ravitaillement communautaire en médicaments antirétroviraux en dehors des structures de santé, récemment introduit, était moins connu (42,2 %). Les dispositifs les plus utilisés étaient l'entretien avec la personne (64 %), le *counseling* (62 %) et le RAVI6M (61,7 %). Les dispositifs les plus appréciés étaient l'approvisionnement semestriel en médicaments antirétroviraux (44,6 %), le comptage des médicaments (10,7 %) et l'entretien avec le patient (10,1 %).

**Conclusion:**

Les dispositifs d'aide à l'observance sont connus et appréciés par les PvVIH. Le ravitaillement tous les 6 mois des médicaments ARV est le plus apprécié. Les politiques communautaires d'approvisionnement en ARV devraient être encouragées.

## Abstract (en)

**Introduction:**

Since the introduction of antiretroviral therapy for HIV in Burkina Faso, several treatment adherence support mechanisms have been implemented to improve outcomes and prevent resistance. Our study aimed to evaluate the knowledge and perceptions of people living with HIV (PLHIV) regarding these mechanisms in the Plateau-Central region.

**Methods:**

We conducted a descriptive study in the Plateau-Central region, which is one of the 13 regions of Burkina Faso. PLHIV were selected as they arrived for their follow-up visits. Information was collected through interviews using a standardized questionnaire. Adherence was calculated based on the participants’ reports. Those who took all their medications in the month prior to the survey were considered adherent. Quantitative variables were calculated using the averages, and qualitative variables were calculated using proportions.

**Results:**

A total of 347 PLHIV were included in the study. Of these, 69% were women, with a mean age of 45.6 ± 12.2 years. The mean treatment follow-up duration was 8.6 ± 5 years. Eighty percent of individuals adhered to treatment (95% CI [75-84]). Nearly all PLHIV (99.7%) were aware of adherence support mechanisms. The most well-known mechanisms were six-month antiretroviral drug supplies (RAVI6M) (71%), discussion groups (69.9%), individual discussion (69.9%), and counseling (64.2%).

The recently introduced community-based antiretroviral drug refilling program outside of health facilities was less well known (42.2%). The most commonly used measures were face-to-face discussion (64%), counseling (62%), and RAVI6M (61.7%). The most appreciated measures were the six-month supply of antiretroviral drugs (44.6%), drug counting (10.7%), and patient interview (10.1%).

**Conclusion:**

PLHIV are familiar with and appreciate adherence support measures. The most appreciated measure is six-monthly refills of ARV drugs. Community-based ARV supply policies should be encouraged.

## Introduction

L'infection par le virus de l'immunodéficience humaine (VIH) demeure un problème mondial de santé publique, avec des implications particulières pour l'Afrique subsaharienne où la prévalence et l'impact de la maladie sont particulièrement élevés [20]. Au Burkina Faso, la prévalence de l'infection par le VIH était estimée à 0,6 % en 2023 chez les 15 à 49 ans [17]. Depuis l'introduction des médicaments antirétroviraux (ARV) contre le VIH, les progrès réalisés ont permis de sauver des millions de vies et d'améliorer la qualité de vie des patients [16]. Des efforts ont été entrepris au fil des années pour faciliter l'accessibilité aux ARV. Depuis 2016, l'Organisation mondiale de la santé (OMS), à travers sa stratégie « *test and treat* », recommande de traiter toute personne dépistée positive au VIH quels que soient le stade clinique et le taux de CD4 [18]. Cette stratégie a permis d'augmenter le nombre de personnes vivant avec le VIH (PvVIH) sous ARV avec une tendance croissante au fil des années. Fin décembre 2023, environ 30,7 millions de personnes dans le monde avaient accès à une thérapie antirétrovirale, contre 7,7 millions [6,7-8 millions] en 2010 [14]. La stratégie de l'OMS souligne l'importance de l'observance, qui reste un défi majeur dans le traitement du VIH. Lorsqu'elle fait défaut, elle expose à un risque accru de résistance virale. Une bonne observance, définie comme d'au moins 95 %, est indispensable pour rendre la charge virale indétectable et garantir l'efficacité des ARV [19]. L'observance aux ARV constitue un des piliers majeurs du succès thérapeutique. La relation entre une bonne observation des soins chez les PvVIH sous traitement antirétroviral et la neutralisation de la charge virale plasmatique a déjà été démontrée [15]. Cependant, celle-ci demeure toujours un défi surtout dans les pays d'Afrique subsaharienne. Le taux d'observance optimal pour rendre une charge virale indétectable n'est pas atteint chez tous les patients sous ARV. Des études réalisées au Cameroun, au Ghana et au Nigeria ont décrit des taux d'observance de 65,2 %, 73 % et 80,6 % respectivement [7, 8, 11].

Pour améliorer l'observance aux ARV, plusieurs stratégies et interventions ont été proposées et mises en place dans les différents pays. Parmi celles-ci, on dénombre l'utilisation des nouvelles technologies de l'information et la communication à travers les SMS de rappel, les appels téléphoniques de rappel, l'implication des agents communautaires, la formation des acteurs de prise en charge, les groupes de discussion, etc. Certains dispositifs ont été plus efficaces, plus adaptés ou mieux perçus que d'autres [2]. Malgré ces dispositifs, l'observance optimale n'est pas atteinte chez tous les PvVIH sous traitement [1]. Leur inefficacité dans certains contextes souligne la nécessité de comprendre les perceptions et les connaissances des acteurs impliqués dans le processus, ainsi que des PvVIH.

L'objectif de la présente étude était de déterminer les connaissances et les perceptions des PvVIH à travers une enquête quantitative sur les dispositifs d'aide à l'observance existant au Burkina Faso à travers le cas de la région du Plateau-Central. Ce projet permettra ainsi de fournir des données pertinentes pour ajuster les politiques de santé publique et améliorer l'observance au traitement, ce qui pourrait avoir un impact direct sur la lutte contre le VIH au Burkina Faso.

## Matériels et méthodes

Nous avons conduit une étude descriptive au mois de novembre 2024. Le site de l’étude a été la région du Plateau-Central, une des 13 régions du Burkina Faso, pays situé en Afrique de l'Ouest. Selon le recensement général de la population et de l'habitat, la population du Burkina Faso était estimée à 20 487 979 habitants en 2019 [9]. Le Plateau-Central est situé au centre du pays et a une population estimée à 1 084 842 habitants en 2023 [13]. Cette région compte trois principaux services de dépistage volontaire (SDV) du VIH et de prise en charge médicale des personnes qui en sont atteintes, répartis dans trois districts sanitaires (Boussé, Ziniaré et Zorgho) et une file active au niveau du Centre hospitalier régional (CHR). Les inclusions au CHR ont débuté en 2022. Notre étude s'est concentrée sur les personnes suivies essentiellement dans les trois districts car celles suivies au CHR avaient majoritairement moins d'un an de suivi, ce qui constituait un critère d'exclusion pour notre étude. Les SDV et de prise en charge médicale des PvVIH sont intégrés dans les activités des services du Centre médical avec antenne chirurgicale (CMA). Le CMA est la formation sanitaire de référence au niveau des districts sanitaires. Les SDV ont été mis en place entre 2006 et 2007 dans la région du Plateau-Central. Au 30 septembre 2024, la file active comptait 2 406 PvVIH, soit 2,91 % de la file active du pays (82 765 PvVIH). La population de cette file active comprend des personnes provenant des zones urbaines et rurales de l'aire sanitaire, et également des zones hors aire sanitaire pour des raisons de proximité de structure de prise en charge et/ou des raisons de confidentialité. La mise sous ARV est actuellement systématique et volontaire pour l'ensemble des personnes dépistées positives. L'organisation est similaire dans les différents centres de la région. Le nombre d'agents est fonction de la taille de la file active et se compose principalement d'un médecin responsable, d'un infirmier et d'un pharmacien. Ce sont des services de jour qui ont des liens fonctionnels avec tous les services susceptibles d'intervenir dans la gestion des PvVIH.

Notre population d’étude concernait les PvVIH suivies dans le cadre de leur traitement dans la région du Plateau-Central.

La taille de l’échantillon a été calculée à partir de la formule standard pour les études descriptives :

**Figure:**
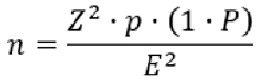


Cette méthode a permis de déterminer la taille adéquate de l’échantillon nécessaire pour garantir la représentativité statistique. Avec un taux d'observance de 75 %, Z à 1,96 et une précision de E à 0,05, un total de 288 sujets était nécessaire pour répondre à nos objectifs. Avec une marge de 20 % donc 72, nous avions besoin de 360 personnes. Nous avons fait un échantillonnage non probabiliste. Les PvVIH ont été recrutées au fur et à mesure qu'elles venaient au rendez-vous de suivi jusqu’à atteindre la taille de l’échantillon.

Les personnes âgées d'au moins 18 ans et suivies dans les files actives dans la région du Plateau-Central depuis au moins un an étaient incluses dans notre étude. Les variables étudiées (âge, genre, durée du traitement, dispositifs d'aide à l'observance connus et utilisés), nombre des visites de suivi, nombre de consultations, etc. sont détaillées dans l'Annexe 1.

Les données ont été collectées *via* des entretiens à l'aide de questionnaires standardisés et saisis dans Kobotoolbox. Dans un premier temps, il a été demandé aux enquêtés de citer les différents dispositifs qu'ils connaissaient. Dans un second temps, les dispositifs non cités ont été évoqués par les enquêteurs. Les dispositifs proposés dans le questionnaire se réfèrent à ceux en vigueur dans le cadre des stratégies mises en œuvre par le Secrétariat permanent du Conseil national de lutte contre le Sida au Burkina Faso. Depuis l'introduction de la thérapie antirétrovirale, ces dispositifs ont évolué au cours des années.

Les données ont été analysées avec le logiciel STATA version 15. Les variables quantitatives ont été décrites en utilisant la moyenne et l’écart-type et les variables qualitatives en utilisant la proportion et l'intervalle de confiance.

Le calcul de l'observance a été fait en se basant sur les déclarations des PvVIH. Ont été considérées comme observant celles qui avaient pris la totalité de leurs médicaments le mois précèdent l'enquête. Le protocole de recherche a été soumis au comité d’éthique de recherche du Burkina Faso (Référence : N° 2024-07-226). Nous avons également obtenu le consentement oral des participants à l’étude. Toutes les dispositions ont été prises pour garder la confidentialité des données des participants à l’étude.

## Résultats

Sur les 360 PvVIH interrogées, les fiches de 347 personnes ont été retenues. Nous avons exclu 13 fiches parce que 4 d'entre elles n'avaient pas le critère d’éligibilité d'au moins 1 an, 6 avaient en réalité 17 ans (et non 18 ans révolus). Pour trois personnes, la variable servant à calculer le taux d'observance n’était pas bien renseignée. L’âge moyen des PvVIH était de 46 ans avec un écart-type de 12 ans. Les extrêmes étaient 19 et 72 ans. Le Tableau I présente les caractéristiques sociodémographiques des PvVIH participant à l’étude. Le temps moyen de suivi pour le traitement était de 9 ans avec un écart-type ±5. Au total, 277 personnes soit 80 % [IC 95 % : 75-84], ont affirmé avoir pris tous leurs médicaments le mois précédant l'enquête, 43 personnes (12 %) ont dit n'avoir pas pris leur traitement une fois, 12 (4 %) ont déclaré n'avoir pas pris leur traitement deux fois, 10 personnes (3 %) ont affirmé n'avoir pas pris leur traitement plus de trois fois et 5 personnes (1 %) ont dit n'avoir pas pris leur traitement le mois dernier. Notre proportion d'observance selon notre définition est de 80 %.

**Tableau I:** Caractéristiques socio-démographiques des PvVIH de la région du Plateau-Central, novembre 2024

Variables	Catégories	Effectif	%
Sexe	Féminin	239	68,8
	Masculin	108	31,1
Nationalité	Burkinabé	339	97,6
	Autres nationalités	8	2,4
Statut matrimonial	En couple	217	62,6
	Célibataires	130	37,4
Profession	Élèves/Étudiants	3	0,9
	Salariés	26	7,7
	Non-Salariés	39	11,6
	Sans emploi formel	277	79,9
Niveau d'instruction	Non alphabétisés	235	69,7
	Alphabétisés	7	2,1
	Primaire	52	15,4
	Secondaire	38	11,3
	Supérieur	5	1,5
Résidence	Boussé	111	32
	Ziniaré	79	22,8
	Zorgho	157	45,2

Au total, 346 PvVIH (99,7 %) avaient déjà entendu parler de l'existence des dispositifs d'aide à l'observance (Tableau II), notamment le ravitaillement des ARV pour une durée de 6 mois - RAVI6M - (71,1 %), l'entretien avec le patient (69,9 %), les groupes de discussion (69,9 %) et le *counseling* (64,2 %). Au total, 345 (99 %) des PvVIH bénéficiaient des activités d'aide à l'observance. Les dispositifs d'aide à l'observance utilisés sont présentés dans le Tableau III.

Parmi les 345 PvVIH, 257 (75 %) ont dit qu'ils se sentaient très bien avec les dispositifs d'aide à l'observance, 82 (24 %) se sentaient bien, 6 (2 %) ont répondu « difficile mais ça va ». L'appréciation des dispositifs d'aide à l'observance est présentée dans le Tableau IV.

Par ailleurs, à travers une question ouverte, nous avons recueilli les suggestions relatives de façon globale au traitement de l'infection par le VIH et ou les dispositifs d'aide à l'observance (Tableau V).

**Tableau II:** Connaissances des dispositifs d'aide à l'observance par les PvVIH dans la région du Plateau-Central, novembre 2024

Dispositifs connus	Oui (%)	Non (%)
Conseils par les pairs	171 (49,4)	175 (50,6)
Formation des acteurs de prise en charge	82 (23,7)	264 (76,3)
Combinaisons thérapeutiques à dose fixe	191 (55,2)	155 (44,8)
Schémas thérapeutiques avec une prise quotidienne	193 (55,8)	153 (44,2)
Accompagnement dans les structures de soins	105 (30,4)	241 (69,7)
Comptage des médicaments	202 (58,4)	144 (41,6)
Pilulier d'une semaine	3 (0,9)	343 (99,1)
Appel téléphonique hebdomadaire	9 (2,6)	337 (97,4)
Sonnerie de rappel quotidienne programmée sur le téléphone de la PvVIH	38 (11)	308 (89)
Envoi de messages (SMS)	8 (2,3)	338 (97,7)
Visites à domicile	70 (20,2)	276 (79,8)
Implication d'un membre de l'entourage dans le traitement	168 (48,6)	178 (51,5)
Groupes de discussion (parole)	242 (69,9)	104 (30,1)
Visites au centre de soins pour des séances de renforcement de l'observance	95 (27,5)	251 (72,5)
Réduction du nombre de médicaments	194 (56,1)	152 (43,9)
Simplification du traitement non ARV pour alléger les prescriptions associées aux ARV	186 (53,8)	160 (46,2)
Recherche de perdus de vue	151 (43,6)	195 (56,4)
Groupes d'auto-support	130 (37,6)	216 (62,4)
Sessions d’éducation thérapeutique	172 (49,7)	174 (50,3)
*Counseling*	222 (64,2)	124 (35,8)
Éducation/causerie éducative	209 (60,4)	137 (39,6)
Entretien avec le patient	242 (69,9)	104 (30,1)
Démonstration	215 (62,1)	131 (37,9)
Ravitaillement des ARV pour une durée de 6 mois	246 (71,1)	100 (28,9)
Ravitaillement communautaire en dehors des structures de santé	146 (42,2)	200 (57,8)

**Tableau III:** Dispositifs d'aide à l'observance utilisés par les PvVIH dans le Plateau-Central, novembre 2024

Dispositifs connus	Oui (%)	Non (%)
Conseils par les pairs	150 (43,5)	195 (56,5)
Formation des acteurs de prise en charge	57 (16,5)	288 (83,5)
Combinaisons thérapeutiques à dose fixe	169 (49)	176 (51)
Schémas thérapeutiques avec une prise quotidienne	174 (50,4)	171 (49,6)
Accompagnement dans les structures de soins	92 (26,7)	253 (73,3)
Comptage des médicaments	177 (51,3)	168 (48,7)
Pilulier	0	347 (100)
Appel téléphonique hebdomadaire	0	347 (100)
Sonnerie de rappel quotidienne programmée sur le téléphone de la PvVIH	31 (9)	314 (91)
Envoi de messages (SMS)	1 (0,3)	344 (99,7)
Visites à domicile	59 (17,1)	286 (82,9)
Implication d'un membre de l'entourage dans le traitement	120 (34,8)	225 (65,2)
Groupes de discussion (parole)	178 (51,6)	167 (48,4)
Visites au centre de soins plus fréquentes que les visites mensuelles	68 (19,7)	277 (80,3)
Réduction du nombre de comprimés	149 (43,2)	196 (56,8)
Simplification du traitement non ARV pour alléger les prescriptions associées aux ARV	168 (48,7)	177 (51,3)
Recherche de perdus de vue	54 (15,6)	291 (84,4)
Groupes d'auto-support	44 (12,8)	301 (87,3)
Sessions d’éducation thérapeutique	71 (20,6)	274 (79,4)
*Counseling*	215 (62,3)	130 (37,7)
Éducation/causerie éducative	169 (49)	176 (51)
Entretien avec le patient	221 (64,1)	124 (36)
Démonstration	201 (58, 3)	144 (41,7)
RAVI6M	213 (61,7)	132 (38,3)
RACODESS*	41 (11,9)	304 (88,1)

* Ravitaillement communautaire en dehors des structures de soins

**Tableau IV:** Classement des dispositifs d'aide à l'observance du mieux apprécié au moins apprécié par les PvVIH dans la région du Plateau-Central, novembre 2024

Dispositifs connus	Oui (%)	Non (%)
RAVI6M	154	44,6
Comptage des médicaments	37	10,7
Entretien avec le patient	35	10,1
Groupes de discussion (parole)	34	9,9
Implication d'un membre de l'entourage dans le traitement	28	8,1
RACODESS*	27	7,8
Éducation/causerie éducative	7	2,0
Appel téléphonique hebdomadaire	5	1,5
Visites à domicile	4	1,2
Recherche de perdus de vue	4	1,2
Sessions d’éducation thérapeutique	4	1,2
Accompagnement dans les structures de soins	3	0,9
Simplification du traitement non ARV	1	0,3
Visites au centre de soins pour des séances de renforcement de l'observance	1	0,3
Conseils par les pairs	1	0,3

* Ravitaillement communautaire en dehors des structures de soins

**Tableau V:** Suggestions formulées par les PvVIH sur les dispositifs d'aide à l'observance dans la région du Plateau-Central, novembre 2024

Suggestions	Effectif
Disposer d'un soutien financier (alimentaire, déplacement)	40
Disposer de médicaments antirétroviraux sous forme injectable	18
Proposer des rendez-vous une fois par an	18
Trouver un médicament qui puisse guérir définitivement	10
Éviter les ruptures de stock	9
Conditionner les comprimés d'ARV actuellement en vrac, sous blister unitaire	7
Disposer de médicaments pour les infections opportunistes	7
Diminuer le volume des comprimés	2
Disposer de boîtes à images	2
Individualiser les rendez-vous	2
Trouver un vaccin contre le VIH	2
Éviter les visites à domicile	1
Bénéficier d'un soutien psychosocial	1
Associer le ravitaillement semestriel au ravitaillement communautaire en dehors des structures de soins	1
Former un médecin spécialisé uniquement dans la prise en charge du VIH dans certaines localités	1
Aucune suggestion	226

## Discussion

Cette étude nous a permis d'estimer la proportion des PvVIH observante aux ARV et d’évaluer leurs connaissances et leurs perceptions sur les dispositifs d'aide existant dans la région du Plateau-Central au Burkina Faso en novembre 2024. Cependant, elle présente quelques insuffisances méthodologiques qui constituent des limites à sa généralisation. Réalisée dans une seule région sur les 13 que comprend le Burkina Faso, la sélection des sujets ne s'est pas faite de façon aléatoire, seule technique pouvant garantir sa représentativité. Par ailleurs, la plupart des questions étant fermées, les informations recueillies ne permettent pas de mieux apprécier les perceptions des personnes sur les stratégies mises en œuvre pour les accompagner dans le traitement de leur maladie.

Les sujets de l’étude étaient constitués majoritairement de femmes. La fréquence du nombre de femmes infectées par le VIH influence les dynamiques de soins et les stratégies d'observance. La peur de la stigmatisation et les pressions sociales, surtout si elles vivent en couple, peuvent freiner l'application de certaines stratégies d'aide à l'observance telles que les visites à domicile ou les ravitaillements communautaires en dehors des structures de soins.

Plus de la moitié des participants vivaient en couple, ce qui peut être un avantage dans le soutien social pour l'observance. Une étude qualitative réalisée en 2017 en Ouganda chez 57 femmes enceintes, conclut que la divulgation du statut au partenaire était un facteur améliorant l'observance [6]. Cependant, la vie en couple peut aussi être un inconvénient pour ceux dont le conjoint n'est pas informé du statut de l'autre. La peur de la divulgation peut constituer un frein pour les visites de suivi, les rendez-vous de ravitaillement et même l'application de certaines stratégies telles que les visites à domicile ou les discussions de groupe. Une grande partie des participants était sans emploi formel et non alphabétisée, ce qui représente un défi socio-économique majeur dans l'adhésion au traitement. Le coût du déplacement pour se rendre au centre de santé pour les rendez-vous de suivi ou dans les lieux de discussion de groupe constitue également un frein.

La durée moyenne de suivi de 8,63 ans traduit une expérience prolongée avec le traitement, mais nécessite d'avoir des dispositifs durables d'aide à l'observance.

La proportion des PvVIH observante n'est pas optimale puisque 1/5 des patients ne le sont pas.

Ce résultat est proche de celui de Mbopi *et al.* dans une étude analytique réalisée en 2012 au Cameroun chez 356 patients [12] mais supérieur à celui retrouvé par Zoungrana *et al.* dans une étude réalisée au Burkina Faso en 2020 chez 1 447 personnes [21]. La proportion élevée des personnes non observantes nécessite donc une étude sur les facteurs associés à l'observance afin de proposer des interventions ciblées pour l'améliorer.

Les dispositifs d'aide à l'observance sont bien connus dans la région du Plateau-Central, même si certains le sont mieux que d'autres. Le RAVI6M, l'entretien avec le patient et les groupes de discussion étaient les mieux connus alors que le pilulier d'une semaine, l'envoi de SMS et l'appel téléphonique hebdomadaire dont l'efficacité a été démontrée par plusieurs auteurs [10, 11] l’étaient beaucoup moins. Cela pourrait s'expliquer par le fait que les acteurs censés mettre en œuvre ces dispositifs ne disposent pas de façon régulière des ressources financières pour acheter les unités de recharge de leur téléphone. Compte tenu de l'efficacité de ces stratégies sur l'observance au traitement antirétroviral, l’État (à travers le ministère de la santé) devrait mieux accompagner les acteurs d'aide à l'observance dans leurs missions. L'utilisation du pilulier doit être promu pour éviter les oublis et respecter les doses.

Le RAVI6M est le dispositif le plus apprécié mais n'est utilisé que chez 60 % des PvVIH. Il s'applique plus particulièrement aux patients stables, c'est-à-dire les PvVIH ayant reçu un traitement pendant au moins un an, qui ne présentent aucune infection opportuniste et qui ont des signes de succès thérapeutique (charge virale inférieures à 1 000 copies/ml). La personne est incluse dans cette stratégie sur la base des résultats de sa dernière charge virale. À défaut, les critères cliniques et immunologiques peuvent être appliqués. Le patient bénéficiant de cette stratégie se rend dans une formation sanitaire tous les six mois pour un réapprovisionnement de son traitement antirétroviral. Après un an de stabilité sous RAVI6M, la visite clinique et la charge virale sont réalisées une fois par an. Les patients peuvent alors se réapprovisionner tous les six mois dans un endroit de réapprovisionnement alternatif, dans la structure sanitaire ou dans la communauté. Ce dispositif présente un grand avantage car les PvVIH ont moins de visites à réaliser : au maximum deux visites par an, contrairement aux autres patients qui ont la contrainte de se rendre tous les deux mois, voire tous les mois dans les formations sanitaires. Cette stratégie leur épargne des coûts de transports et des pertes de temps. Grâce à la diminution des visites aux centres de santé, les patients RAVI6M sont moins exposés au comportement de discrimination ou de stigmatisation par l'entourage et les autres usagers des formations sanitaires. Un autre avantage est la réduction de la charge de travail pour les agents de santé, les PvVIH se rendant moins souvent en consultation dans les formations sanitaires. Des dispositions devraient donc être prises pour stabiliser les patients afin qu'ils puissent tous bénéficier de cette stratégie au-delà d'un an de suivi. Plusieurs études ont démontré que les rendez-vous espacés étaient appréciés par les patients et permettaient de les maintenir aux soins [3, 4].

Le ravitaillement communautaire en dehors des structures de soins, une stratégie nouvellement mise en œuvre pour améliorer l'observance, arrive en dixième position des mesures connues. La dispensation communautaire des ARV permet d'atteindre les populations qui ne peuvent pas aller dans les formations sanitaires pendant les heures normales de travail pour plusieurs raisons (financières, géographiques, sociales, religieuses). Cette stratégie n'est pas très appréciée probablement parce que certaines personnes ont peur que des membres de la communauté découvrent leur statut, notamment les voisins et la famille [5]. D'autres ont plus confiance dans les agents de santé que dans les acteurs communautaires, tant sur le plan professionnel que confidentiel. Certaines personnes peuvent également douter de la qualité des médicaments qui sont dispensés par les acteurs communautaires.

## Conclusion

Toutes les personnes suivies dans les files actives ne sont pas observantes. Les dispositifs d'aide à l'observance sont divers, connus et appréciés par les PvVIH et les acteurs d'aide à l'observance même si certains dispositifs le sont plus que d'autres. Le ravitaillement semestriel est le plus connu et le plus apprécié même s'il n'est pas utilisé par tous. Des dispositions devraient être prises par les programmes de lutte contre le VIH pour améliorer l’état clinique de toutes les PvVIH afin que celles qui le souhaitent puissent bénéficier de cette stratégie. Ceci contribuerait probablement à atteindre l'observance optimale chez toutes les PvVIH.

## Source de financement

Cette étude n'a reçu aucun financement.

## Contribution des auteurs et autrices

Wedminère Noélie ZOUNGRANA-YAMEOGO est le concepteur de l’étude. Elle a analysé et interprété les résultats et a écrit la première ébauche. Les données ont été collectées et saisies par Dominique Hélène Laurel YABRE. Dominique Hélène Laurel YAMBRE, Fidèle BAKIONO, Toussaint COMPAORE, Arielle Rita BELEM, David KANGOYE, Christian YONLI et Ouo Mireille COULIBALY ont lu le premier document et ont fait des observations. Koiné Maxime DRABO a supervisé les travaux de cette étude et a donné son accord pour sa publication.

## Liens d'intérêts

Les auteurs ne déclarent ne pas avoir de liens d'intérêt.

## Annexe 1 : Fiche de collecte

### Questionnaire pour les PvVIH sur les dispositifs d'aide à l'observance

Je suis madame, monsieur mademoiselle… et je travaille dans le cadre de la recherche d'une étude intitulée « Connaissances, perceptions des personnes vivant avec le VIH sur les dispositifs d'aide à l'observance existant au Burkina Faso ». Cette étude est dirigée par le Dr. YAMEOGO/ZOUNGRANA W. Noélie sous la supervision du Pr DRABO Maxime.

Nous avons sélectionné des sites de prise en charge de PvVIH dans le Plateau-Central dont nous proposons aux PvVIH de participer à cette étude. Cette participation se fait à travers la soumission à un questionnaire.

Les résultats de cette étude seront présentés aux décideurs, chercheurs, acteurs de terrain. Mais il ne sera nullement fait mention de votre identité ou de toute déclaration susceptible de vous reconnaître.

Vous n’êtes pas obligé de prendre part à cette étude. Si vous refusez d'y participer, cela n'affectera nullement les soins que vous recevez. Nous prendrons toutes les dispositions nécessaires pour assurer la confidentialité de vos informations.

Vous pouvez vous retirer de l’étude à tout moment. Votre décision de vous retirer de l’étude n'affectera en rien les soins que vous recevez.

Si vous avez des questions, vous pouvez les poser maintenant ou plus tard. Si vous souhaitez le faire plus tard, les personnes que vous pouvez contacter sont les suivantes :

Contact Dr Zoungrana

**Table:**

Êtes-vous d'accord pour participer à l’étude ?		Oui /__/ Non /__/
N°	Questions	Réponses
Les informations personnelles du PvVIH		
Q1	Date de collecte	/_/2024
Q2	N* d'affiliation	
Q3	Numéro de téléphone	
Q4	Âge	
Q5	Sexe	F/_/ M/_/
Q6	Nationalité	Burkinabè/_/ Autre/_/
Q7	Statut matrimonial	Célibataire/_/ En couple/_/
Q8	Profession	Sans emploi/_/ Élève/Étudiant/_/ Salarié : Public/_/Privé/_/ Non salarié/_/
Q9	Niveau d'instruction	Aucun/_/ Alphabétisé/_/ Primaire/_/ Secondaire/_/ Supérieur/_/
Q10	Résidence	Boussé/_/ Ziniaré/_/ Zorgho/_/
Informations sur le VIH		
Q11	Depuis combien de temps savez-vous que vous êtes une PvVIH ?	
Q12	Comment vous sentez-vous depuis ce moment ?	Très mal/_/ Mal/_/ Difficile, mais ça va/_/
Q13	Vous suivez un traitement ARV ? Si oui, combien de temps en année ?	
Q14	Si oui, comment vous sentez vous avec ce traitement ?	Très bien /_/ Bien/_/ Assez bien/_/ Mal/_/ Très mal/_/ Difficile, mais ça va/_/
Q15	Combien de fois vous est-il arrivé de ne pas prendre votre traitement antirétroviral dans le mois passé ?	Jamais/_/Une fois/_/ Trois fois plus/_/ Deux fois/_/ Je n'ai pas pris mon traitement le mois dernier/_/
Les connaissances sur les dispositifs d'aide à l'observance		
Q16	Avez-vous entendu parler des dispositifs d'aide à l'observance ?	Oui/_/Non/_/
Q17	Si oui, parmi les dispositifs suivants, desquels avez-vous entendu parler ?	
	Les conseils par les pairs	Oui/_/ Non/_/
	La formation des acteurs de la prise en charge	Oui/_/ Non/_/
	Les combinaisons thérapeutiques à dose fixe	Oui/_/ Non/_/
	Les schémas thérapeutiques avec une prise quotidienne	Oui/_/ Non/_/
	L'accompagnement dans les structures de soins	Oui/_/ Non/_/
	Le comptage des médicaments	Oui/_/ Non/_/
	Le pilulier d'une semaine	Oui/_/ Non/_/
	L'appel téléphonique hebdomadaire	Oui/_/ Non/_/
	La sonnerie de rappel quotidienne programmée sur le téléphone de la PvVIH	Oui/_/ Non/_/
	L'envoi de message (SMS)	Oui/_/ Non/_/
	Les visites à domicile	Oui/_/ Non/_/
	L'implication d'un membre de l'entourage dans le traitement	Oui/_/ Non/_/
	Les groupes de parole	Oui/_/ Non/_/
	Les visites au centre de soins plus fréquentes que les visites mensuelles, pour des séances de renforcement de l'observance	Oui/_/ Non/_/
	La réduction du nombre de comprimés	Oui/_/ Non/_/
	La simplification du traitement non ARV pour alléger les prescriptions annexées aux ARV	Oui/_/ Non/_/
	La recherche de perdus de vue	Oui/_/ Non/_/
	Les groupes d'auto-support	Oui/_/ Non/_/
	Les sessions d’éducation thérapeutique	Oui/_/ Non/_/
	Le *counseling*	Oui/_/ Non/_/
	L’éducation/causerie éducative	Oui/_/ Non/_/
	L'entretien avec le patient	Oui/_/ Non/_/
	La démonstration	Oui/_/ Non/_/
	RAVI6M	Oui/_/ Non/_/
	RACODess	Oui/_/ Non/_/
Q18	Bénéficiez-vous d'activité d'aide à l'observance ?	Oui/_/ Non/_/ Si non, fin du questionnaire
Q19	Parmi les dispositifs suivants lesquels utilisez-vous ?	
	Les conseils par les pairs	Oui/_/ Non/_/
	La formation des acteurs de la prise en charge	Oui/_/ Non/_/
	Les combinaisons thérapeutiques à dose fixe	Oui/_/ Non/_/
	Les schémas thérapeutiques avec une prise quotidienne	Oui/_/ Non/_/
	L'accompagnement dans les structures de soins	Oui/_/ Non/_/
	Le comptage des médicaments	Oui/_/ Non/_/
	Le pilulier d'une semaine	Oui/_/ Non/_/
	L'appel téléphonique hebdomadaire	Oui/_/ Non/_/
	La sonnerie de rappel quotidienne programmée sur le téléphone de la PvVIH	Oui/_/ Non/_/
	L'envoi de messages (SMS)	Oui/_/ Non/_/
	Les visites à domicile	Oui/_/ Non/_/
	L'implication d'un membre de l'entourage dans le traitement	Oui/_/ Non/_/
	Les groupes de parole	Oui/_/ Non/_/
	Les visites au centre de soins plus fréquentes que les visites mensuelles, pour des séances de renforcement de l'observance	Oui/_/ Non/_/
	La réduction du nombre de comprimés	Oui/_/ Non/_/
	La simplification du traitement non ARV pour alléger les prescriptions annexées aux ARV	Oui/_/ Non/_/
	La recherche de perdus de vue	Oui/_/ Non/_/
	Les groupes d'auto-support	Oui/_/ Non/_/
	Sessions d’éducation thérapeutique	Oui/_/ Non/_/
	Le *counseling*	Oui/_/ Non/_/
	L'éducation/la causerie éducative	Oui/_/ Non/_/
	L'entretien avec le patient	Oui/_/ Non/_/
	La démonstration	Oui/_/ Non/_/
	Le RAVI6M	Oui/_/ Non/_/
	Le RACODess	Oui/_/ Non/_/
Perceptions des PvVIH sur les dispositifs d'aide		
Q20	Comment vous sentez-vous avec l'utilisation de ces dispositifs d'aide à l'observance ?	Mal/_/ Difficile, mais ça va/_/ Bien/_/ Très bien/_/
Q21	Quel dispositif vous permet de mieux suivre votre traitement ?	
Q22	Avez-vous des suggestions pour l'amélioration de ces dispositifs pour une meilleure prise en charge de votre traitement ? si oui faites part	
