# Supplementary material for: Knowledge and perceptions of people living with HIV regarding adherence support mechanisms in treatment centers in the Plateau-Central region of Burkina Faso, November 2024
Source: Med Trop Sante Int. 2025 May 12;5(2):mtsi.v5i2.2025.643. [Article in French] doi: 10.48327/mtsi.v5i2.2025.643 (PMC12308576; doi:10.48327/mtsi.v5i2.2025.643)
Supplement: Related texts

# Research article: Knowledge and perceptions of people living with HIV regarding adherence support mechanisms in treatment centers in the Plateau-Central region of Burkina Faso, November 2024

## Introduction

Infection with the human immunodeficiency virus (HIV) remains a significant global public health concern, particularly in sub-Saharan Africa, where the prevalence and impact of the disease are high [20]. In Burkina Faso, the HIV infection prevalence was estimated at 0.6% among 15- to 49-year-old in 2023 [17]. Since the introduction of antiretroviral drugs (ARVs) for treating HIV, millions of lives have been saved, and patients’ quality of life has improved [16]. Efforts have been made over the years to improve access to ARVs. Since 2016, the World Health Organization (WHO) has recommended treating anyone who tests positive for HIV, regardless of clinical stage or CD4 count, through its “test and treat” strategy [18]. This strategy has increased the number of people living with HIV (PLHIV) on ARVs, and this number has grown over the years. As of December 2023, approximately 30.7 million people in the world had access to ARVs, up from 7.7 million in 2010 [6,7-8 millions] in 2010 [14]. The WHO strategy emphasizes the importance of adherence, which remains a major challenge in HIV treatment. Poor adherence increases the risk of viral resistance. Good adherence, defined as taking at least 95% of prescribed doses, is essential to achieving an undetectable viral load and ensuring the effectiveness of ARVs [19]. Adherence to ARVs is one of the major pillars of treatment success. The relationship between good adherence to care among PLHIV on antiretroviral treatment and the neutralization of plasma viral load has already been demonstrated [15]. However, this remains challenging, particularly in sub-Saharan African countries. The optimal adherence rate for achieving an undetectable viral load is not reached by all patients on ARVs. Studies in Cameroon, Ghana, and Nigeria reported adherence rates of 65.2%, 73%, and 80.6%, respectively [7, 8, 11]. Several strategies and interventions have been proposed and implemented in various countries to improve ARV adherence. These include using new information and communication technologies, such as text message reminders and reminder phone calls; involving community workers; training healthcare providers; and facilitating discussion groups. Some of these measures have been more effective, appropriate, or well-received than others [2]. Despite these measures, however, optimal adherence is not achieved among all PLHIV on treatment [1]. Their ineffectiveness in certain contexts underscores the importance of understanding the perceptions and knowledge of those involved in the process, including PLHIV. This study aimed to assess PLHIV's knowledge and perceptions of existing adherence support mechanisms in Burkina Faso's Plateau-Central region. This project will thus provide relevant data to adjust public health policies and improve treatment adherence, which could directly impact the fight against HIV in Burkina Faso.

## Materials and methods

We conducted a descriptive study in November 2024. The study site was the Plateau-Central region, which is one of thirteen regions in Burkina Faso, a country located in West Africa. According to the 2019 general population and housing census, Burkina Faso's population was estimated at 20,487,979 [9]. The Plateau-Central region is located in the center of Burkina Faso and had an estimated population of 1,084,842 in 2023 [13]. The region offers three main HIV voluntary screening services (VSS) and medical care for people living with HIV. They are spread across three health districts (Boussé, Ziniaré, and Zorgho), as well as an active list at the Regional Hospital Center (RHC). Enrollment at the RHC began in 2022. Our study focused on individuals primarily monitored in the three districts because those monitored at the RHC had mostly been monitored for less than a year, which was an exclusion criterion for our study. VSS and medical care services for PLHIV are integrated into the activities of the Medical Centers with Surgical Unit, which are the referral health facilities at the district level. VSS were established in the Plateau-Central region between 2006 and 2007. As of September 30, 2024, 2,406 PLHIV were on the active list, representing 2.91% of the country's active list of 82,765 PLHIV. This active list includes people from urban and rural areas of the health zone, as well as from outside the health zone, due to proximity to care facilities and/or confidentiality reasons. ARV treatment is currently systematic and voluntary for all individuals who test positive. The organization is similar in the different centers in the region. Staff numbers depend on the size of the active list and consist mainly of a doctor in charge, a nurse, and a pharmacist. These are day services with functional links to all services involved in managing PLHIV.

Our study population consisted of PLHIV who were receiving treatment in the Plateau-Central region.

We calculated the sample size using the standard formula for descriptive studies.

This method determined the appropriate sample size to ensure statistical representativeness. With a 75% compliance rate, a Z value of 1.96, and an E value of 0.05, we needed a total of 288 subjects to meet our objectives. With a margin of error of 20%, or 72, we needed 360 people.

We used non-probability sampling. PLHIV were recruited as they came to their follow-up appointments until the sample size was reached.

Our study included people aged 18 years or older who had been in active care in the Plateau-Central region for at least one year. The studied variables (age, gender, duration of treatment, known and used adherence support measures, number of follow-up visits, number of consultations, etc.) are detailed in Appendix 1.

Data were collected via interviews using standardized questionnaires and entered into KoboToolbox. Initially, respondents were asked to name the various mechanisms they were aware of. Mechanisms not mentioned were then raised by the interviewers. The measures proposed in the questionnaire refer to those in force as part of the strategies implemented by the Permanent Secretariat of the National Council for the Fight Against AIDS in Burkina Faso. These measures have evolved over the years since the introduction of antiretroviral therapy.

The data were analyzed using Stata version 15 software. Quantitative variables were described using the mean and standard deviation, while qualitative variables were described using proportions and confidence intervals.

Adherence was calculated based on PLHIV's self-reports. Those who took all their medication in the month before the survey were considered compliant.

We submitted the research protocol to the Burkina Faso Research Ethics Committee (reference no. 2024-07-226). We obtained verbal consent from the study participants. All measures were taken to maintain the confidentiality of the participants’ data.

## Results

Of the 360 PLHIV interviewed, the records of 347 people were retained. Thirteen records were excluded: four did not meet the one-year eligibility criterion, and six were actually 17 years old (not 18). For three individuals, the variable used to calculate the adherence rate was not well documented. The average age of the PLHIV was 46 years, with a standard deviation of 12 years. The ages ranged from 19 to 72 years old. Table I presents the sociodemographic characteristics of the PLHIV who participated in the study.

**Table I:** Socio-demographic characteristics of PLHIV in the Central Plateau region, November 2024

Variables	Categories	Headcount	%
Gender	Female	239	68,8
	Male	108	31,1
Nationality	Burkinabe	339	97,6
	Other nationalities	8	2,4
Marital status	In a relationship	217	62,6
	Single	130	37,4
Occupation	Students	3	0,9
	Employed	26	7,7
	Freelance	39	11,6
	Without formal job	277	79,9
Level of education	Illiterate	235	69,7
	Literate	7	2,1
	Primary	52	15,4
	Secondary	38	11,3
	Higher	5	1,5
Residence	Boussé	111	32
	Ziniaré	79	22,8
	Zorgho	157	45,2

The average treatment follow-up time was nine years, with a standard deviation of ±5.

A total of 277 people (80%; 95% CI: 75%-84%) reported taking all their medication in the month prior to the survey. Forty-three people (12%) said they had missed their treatment once; 12 people (4%) said they had missed it twice; 10 people (3%) said they had missed it more than three times; and five people (1%) said they had not taken it in the last month. According to our definition, our adherence rate is 80%.

A total of 346 PLHIV (99.7%) had heard of adherence support mechanisms, including six-month ARV refills (RAVI6M) (71.1%), patient discussion (69.9%), discussion groups (69.9%), and counseling (64.2%) (Table II). A total of 345 PLHIV (99%) benefited from these activities. The adherence support mechanisms used are presented in Table III. Of the 345 PLHIV, 257 (75%) said they felt very comfortable with the support measures, 82 (24%) felt comfortable, and six (2%) responded “difficult but okay.” The assessment of the support measures is presented in Table IV.

Additionally, we collected suggestions related to HIV treatment and/or adherence support mechanisms in general through an open-ended question (Table V).

**Table II:** Knowledge of adherence support mechanisms among PLHIV in the Plateau-Central region, November 2024

Known measures	Yes (%)	No (%)
Peer counseling	171 (49,4)	175 (50,6)
Training for healthcare providers	82 (23,7)	264 (76,3)
Fixed-dose combination therapies	191 (55,2)	155 (44,8)
Daily treatment regimens	193 (55,8)	153 (44,2)
Support in healthcare facilities	105 (30,4)	241 (69,7)
Medication counting	202 (58,4)	144 (41,6)
Weekly pill box	3 (0,9)	343 (99,1)
Weekly phone call	9 (2,6)	337 (97,4)
Daily reminder alarm set on the PLHIV's phone	38 (11)	308 (89)
Text messages (SMS)	8 (2,3)	338 (97,7)
Home visits	70 (20,2)	276 (79,8)
Involvement of a family member in treatment	168 (48,6)	178 (51,5)
Discussion groups (talking)	242 (69,9)	104 (30,1)
Visits to the healthcare center for adherence reinforcement sessions	95 (27,5)	251 (72,5)
Reduction in the number of drugs	194 (56,1)	152 (43,9)
Simplification of non-ARV treatment to reduce prescriptions associated with ARVs	186 (53,8)	160 (46,2)
Search for lost to follow-up	151 (43,6)	195 (56,4)
Self-support groups	130 (37,6)	216 (62,4)
Therapeutic education sessions	172 (49,7)	174 (50,3)
Counseling	222 (64,2)	124 (35,8)
Education/educational talks	209 (60,4)	137 (39,6)
Patient interviews	242 (69,9)	104 (30,1)
Demonstrations	215 (62,1)	131 (37,9)
Six-month supply of ARVs	246 (71,1)	100 (28,9)
Community supply outside health facilities	146 (42,2)	200 (57,8)

**Table III:** Adherence support measures used by PLHIV in the Plateau-Central, November 2024

Known measures	Yes (%)	No (%)
Peer counseling	150 (43,5)	195 (56,5)
Training for healthcare providers	57 (16,5)	288 (83,5)
Fixed-dose combination therapies	169 (49)	176 (51)
Daily treatment regimens	174 (50,4)	171 (49,6)
Support in healthcare facilities	92 (26,7)	253 (73,3)
Medication counting	177 (51,3)	168 (48,7)
Pill box	0	347 (100)
Weekly phone call	0	347 (100)
Daily reminder alarm set on the PLHIV's phone	31 (9)	314 (91)
Text messages (SMS)	1 (0,3)	344 (99,7)
Home visits	59 (17,1)	286 (82,9)
Involvement of a family member in treatment	120 (34,8)	225 (65,2)
Discussion groups (talking)	178 (51,6)	167 (48,4)
More frequent visits to the healthcare center than monthly visits	68 (19,7)	277 (80,3)
Reduction in the number of tablets	149 (43,2)	196 (56,8)
Simplification of non-ARV treatment to reduce prescriptions associated with ARVs	168 (48,7)	177 (51,3)
Search for lost to follow-up	54 (15,6)	291 (84,4)
Self-support groups	44 (12,8)	301 (87,3)
Therapeutic education sessions	71 (20,6)	274 (79,4)
Counseling	215 (62,3)	130 (37,7)
Education/educational talks	169 (49)	176 (51)
Patient interviews	221 (64,1)	124 (36)
Demonstrations	201 (58, 3)	144 (41,7)
RAVI6M	213 (61,7)	132 (38,3)
RACODESS*	41 (11,9)	304 (88,1)

* Community refilling outside healthcare facilities

**Table IV:** Ranking of adherence support measures from most to least appreciated by PLHIV in the Plateau-Central region, November 2024

Known measures	Yes (%)	No (%)
RAVI6M	154	44,6
Medication counting	37	10,7
Patient interviews	35	10,1
Discussion groups (talking)	34	9,9
Involvement of a family member in treatment	28	8,1
RACODESS*	27	7,8
Education/educational talks	7	2,0
Weekly phone call	5	1,5
Home visits	4	1,2
Search for lost to follow-up	4	1,2
Therapeutic education sessions	4	1,2
Support in healthcare facilities	3	0,9
Simplification of non-ARV treatment	1	0,3
Visits to the healthcare center for adherence reinforcement sessions	1	0,3
Peer counseling	1	0,3

Community refilling outside healthcare facilities

**Table V:** Suggestions of PLHIV for the measures to help observance in the Plateau-Central region, November 2024

Suggestions	Headcount
Have financial support (food, transportation)	40
Have access to injectable antiretroviral drugs	18
Offer appointments once a year	18
Find a drug that can cure the disease permanently	10
Avoid stock shortages	9
Pack ARV tablets currently in bulk, in individual blister packs	7
Have medication available for opportunistic infections	7
Reduce the size of the tablets	2
Have picture cards available	2
Individualize appointments	2
Find a vaccine against HIV	2
Avoid home visits	1
Provide psychosocial support	1
Combine six-monthly refills with community refills outside healthcare facilities	1
Train a doctor specializing solely in HIV care in certain localities	1
No suggestions	226

## Discussion

This study allowed us to estimate the proportion of PLHIV who adhere to ARV treatment and assess their knowledge of and perceptions about existing support mechanisms in the Plateau-Central region of Burkina Faso in November 2024. However, the study has some methodological shortcomings that limit its generalizability. First, it was conducted in only one of Burkina Faso's 13 regions, and the subjects were not randomly selected, which is the only technique that can guarantee representativeness. Furthermore, most of the questions were closed-ended, so the collected information does not allow for a better assessment of people's perceptions of the implemented support strategies for their disease treatment.

The study subjects were mainly women. The high prevalence of HIV infection among women influences care dynamics and adherence strategies. Fear of stigmatization and social pressures, especially among those living with a partner, can hinder the implementation of certain support strategies, such as home visits or community refills outside of healthcare facilities.

More than half of the participants lived with a partner, which can be an advantage in the social support for adherence. A 2017 qualitative study conducted in Uganda among 57 pregnant women concluded that disclosing one's status to a partner improved adherence [6]. However, living as a couple can also be disadvantageous for those whose partners are unaware of their status. Fear of disclosure can hinder follow-up visits, refill appointments, and the implementation of strategies such as home visits and group discussions. Many participants were without a formal job and illiterate, representing a major socioeconomic challenge to treatment adherence. The cost of traveling to the health center for follow-up appointments or group discussions is also a barrier. The average follow-up duration of 8.63 years indicates long-term treatment experience, but requires sustainable support mechanisms for adherence. The proportion of PLHIV who are adherent is not optimal since one-fifth of patients are not adherent. This result is similar to that of Mbopi *et al.* study conducted in Cameroon in 2012 among 356 patients, but higher than that found by Zoungrana *et al.* study conducted in Burkina Faso in 2020 among 1,447 individuals [21]. Therefore, the high proportion of non-adherent individuals requires a study of the factors associated with adherence in order to propose targeted interventions to improve it.

Although some are better known than others, adherence support mechanisms are well known in the Plateau-Central region. The RAVI6M, patient interviews, and focus groups were the best known. In contrast, the weekly pill box, text messaging, and weekly phone calls, the effectiveness of which has been demonstrated by several authors, were much less well-known [10, 11]. This could be explained by the fact that those responsible for implementing these measures lack regular financial resources to purchase phone recharge units. Given these strategies’ effectiveness on adherence to antiretroviral treatment, the government (through the Ministry of Health) should provide better support to those involved in help for adherence to treatment.

The use of pill dispensers should be promoted to prevent missed doses and ensure compliance. The RAVI6M is the most popular device, yet only 60% of PLHIV use it. It is particularly applicable to stable patients, that is, PLHIV who have been on treatment for at least one year, have no opportunistic infections, and show signs of treatment success, such as a viral load below 1,000 copies/mL. A person is included in this strategy based on their most recent viral load results. If these results are unavailable, clinical and immunological criteria may be applied. Patients who benefit from this strategy visit a health facility every six months to replenish their antiretroviral treatment. After one year of stability on RAVI6M, clinical visits and viral load testing are performed annually. Patients can then obtain a refill every six months at an alternative refill site, either at the health facility or in the community. This system has a major advantage in that PLHIV have fewer visits to make, with a maximum of two visits per year. This is in contrast to other patients, who are required to visit health facilities every two months or even every month. This saves them transportation costs and time. Due to the reduced number of visits to health centers, RAVI6M patients are less exposed to discrimination or stigmatization by their families and other health facility users. Another advantage is that health workers have a reduced workload because PLHIV visit health facilities less often. Therefore, measures should be taken to stabilize patients so they can all benefit from this strategy beyond one year of follow-up. Several studies have shown that spaced appointments are appreciated by patients and help keep them in care [3, 4].

Community refilling outside of healthcare facilities, a newly implemented strategy to improve adherence, is tenth on the list of known measures.

Community dispensing of ARVs makes it possible to reach populations who cannot go to healthcare facilities during normal working hours for various reasons, such as financial, geographical, social, or religious issues. This strategy is not very popular, probably because some people are afraid that community members, particularly neighbors and family, will discover their status [5]. Others have more confidence in healthcare workers than community members, both professionally and in terms of confidentiality. Some people may also doubt the quality of drugs dispensed by community members.

## Conclusion

Not everyone on active list is adherent. Although some mechanisms are more popular than others, adherence support mechanisms are diverse, known, and appreciated by PLHIV and adherence support actors. Six-month refills are the most well-known and appreciated, though not everyone uses them. HIV programs should make provisions to improve the clinical status of all PLHIV allowing those who wish to do so to benefit from this strategy. This would likely contribute to achieving optimal adherence among all PLHIV.

## Funding

This study received no funding.

## Authors’ contributions

Wedminère Noélie ZONGRANA-YAMEOGO designed the study. She analyzed and interpreted the results and wrote the first draft. Dominique Hélène Laurel YAMBRE collected and entered the data. Dominique Hélène Laurel YABRE, Fidèle BAKIONO, Toussaint COMPAORE, Arielle Rita BELEM, David KANGOYE, Christian YONLI and Ouo Mireille COULIBALY read the first draft and provided comments. Koiné Maxime DRABO supervised the study and approved it for publication.

## Conflicts of interest

The authors declare no competing interests.

## Appendix 1: Data collection form

### Questionnaire for PLHIV on adherence support services

My name is Ms., Mr., Miss… and I am working on a research study entitled “Knowledge and perceptions of people living with HIV on existing adherence support services in Burkina Faso.” This study is led by Dr. YAMEOGO/ZOUNGRANA W. Noélie under the supervision of Prof. DRABO Maxime.

We have selected PLHIV care sites in the PlateauCentral where we are inviting PLHIV to participate in this study. Participation involves completing a questionnaire.

The results of this study will be presented to decision-makers, researchers, and field workers. However, no mention will be made of your identity or any statements that could identify you.

You are not obliged to take part in this study. If you refuse to participate, this will not affect the care you receive. We will take all necessary measures to ensure the confidentiality of your information.

You can withdraw from the study at any time. Your decision to withdraw from the study will not affect the care you receive.

If you have any questions, you can ask them now or later. If you wish to do so later, you can contact the following people:

Contact Dr. Zoungrana

**Table:**

Do you agree to participate in the study?		Yes /__/ No /__/
N°	Questions	Réponses
Personal information of PLHIV		
Q1	Date of collection	//2024
Q2	Affiliation number	
Q3	Phone number	
Q4	Age	
Q5	Gender	F/_/ M/_/
Q6	Nationality	Burkinabe/_/ Other/_/
Q7	Marital status	Single/_/ In a relationship /_/
Q8	Profession	Unemployed /_/ Student /_/ Employee: Public/_/Private/_/ Freelance /_/
Q9	Level of education	None/_/ Literate/_/ Primary/_/ Secondary/_/ Higher/_/
Q10	Place of residence	Boussé/_/ Ziniaré/_/ Zorgho/_/
Information about HIV		
Q11	How long have you known that you are PLHIV?	
Q12	How have you felt since then?	Very bad/_/ Bad/_/ Difficult, but okay/_/
Q13	Are you receiving ARV treatment? If yes, for how many years?	
Q14	If yes, how do you feel about this treatment?	Very good /_/ Good/_/ Fairly good/_/ Bad/_/ Very bad/_/ Difficult, but okay/_/
Q15	How many times have you missed taking your antiretroviral treatment in the past month?	Never/_/ Once/_/ Three times or more/_/ Twice/_/ I did not take my treatment last month/_/
Knowledge of adherence support mechanisms		
Q16	Have you heard of adherence support mechanisms?	Yes/_/No/_/
Q17	If yes, which of the following mechanisms have you heard of?	
	Peer counseling	Yes/_/ No/_/
	Training for healthcare professionals	Yes/_/ No/_/
	Fixed-dose combination therapy	Yes/_/ No/_/
	Daily dosing regimens	Yes/_/ No/_/
	Support in healthcare facilities	Yes/_/ No/_/
	Medication counting	Yes/_/ No/_/
	Weekly pill box	Yes/_/ No/_/
	Weekly phone call	Yes/_/ No/_/
	Daily reminder alarm set on the PLHIV's phone	Yes/_/ No/_/
	Text messaging (SMS)	Yes/_/ No/_/
	Home visits	Yes/_/ No/_/
	Involvement of a family member in treatment	Yes/_/ No/_/
	Support groups	Yes/_/ No/_/
	Visits to the healthcare center more frequent than monthly visits, for compliance reinforcement session	Yes/_/ No/_/
	Reduction in the number of tablets	Yes/_/ No/_/
	Simplification of non-ARV treatment to reduce prescriptions associated with ARVs	Yes/_/ No/_/
	Search for lost to follow-up	Yes/_/ No/_/
	Self-support groups	Yes/_/ No/_/
	Therapeutic education sessions	Yes/_/ No/_/
	Counseling	Yes/_/ No/_/
	Education/educational talks	Yes/_/ No/_/
	Patient interviews	Yes/_/ No/_/
	Demonstration	Yes/_/ No/_/
	RAVI6M	Yes/_/ No/_/
	RACODess	Yes/_/ No/_/
Q18	Do you receive any help with adherence?	Yes/_/ No/_/ If not, end of questionnaire
Q19	Which of the following do you use?	
	Peer counseling	Oui/_/ Non/_/
	Training for healthcare professionals	Oui/_/ Non/_/
	Fixed-dose combination therapy	Oui/_/ Non/_/
	Daily dosing regimens	Oui/_/ Non/_/
	Support in healthcare facilities	Oui/_/ Non/_/
	Medication counting	Oui/_/ Non/_/
	Weekly pill box	Oui/_/ Non/_/
	Weekly phone call	Oui/_/ Non/_/
	Daily reminder alarm set on the PLHIV's phone	Oui/_/ Non/_/
	Text messaging (SMS)	Oui/_/ Non/_/
	Home visits	Oui/_/ Non/_/
	Involvement of a family member in treatment	Yes/_/ No/_/
	Support groups	Yes/_/ No/_/
	Visits to the healthcare center more frequent than monthly visits, for compliance reinforcement session	Yes/_/ No/_/
	Reduction in the number of tablets	Yes/_/ No/_/
	Simplification of non-ARV treatment to reduce prescriptions associated with ARVs	Yes/_/ No/_/
	Search for lost to follow-up	Yes/_/ No/_/
	Self-support groups	Yes/_/ No/_/
	Therapeutic education sessions	Yes/_/ No/_/
	Counseling	Yes/_/ No/_/
	Education/educational talks	Yes/_/ No/_/
	Patient interviews	Yes/_/ No/_/
	Demonstration	Yes/_/ No/_/
	RAVI6M	Yes/_/ No/_/
	RACODess	Yes/_/ No/_/
PLHIV perceptions of support mechanisms		
Q20	How do you feel about using these adherence support mechanisms?	Bad/_/ Difficult, but okay/_/ Good/_/ Very good/_/
Q21	Which device helps you to follow your treatment better?	
Q22	Do you have any suggestions for improving these devices to help you manage your treatment better? If so, please share them.
